# The Role of Cartilage Stem/Progenitor Cells in Cartilage Repair in Osteoarthritis

**DOI:** 10.2174/1574888X17666221006113739

**Published:** 2023-05-29

**Authors:** Ning Hu, Jingwen Qiu, Bo Xu, Shunhao Zhang, Zijian Guo, Jing Xie, Wenbin Yang

**Affiliations:** 1 State Key Laboratory of Oral Disease, National Clinical Research Center for Oral Disease, West China Hospital of Stomatology, Sichuan University, Chengdu, Sichuan Province, China;; 2 Department of Stomatology, Panzhihua Central Hospital, Panzhihua City, Sichuan Province, China;; 3 State Key Laboratory of Oral Diseases, National Clinical Research Center for Oral Diseases, Department of Medical Affairs, West China Hospital of Stomatology, Sichuan University, Chengdu, China

**Keywords:** Cartilage stem cell, cartilage progenitor cell, osteoarthritis, cartilage, regenerative therapy, stem cell therapy

## Abstract

Osteoarthritis (OA) is a degenerative joint disease characterized by the loss of cartilage, which seriously affects the quality of patient's life and may even cause permanent sequelae. The treatment of OA is diversified, mostly limited to relieving clinical symptoms. Less invasive treatments that can cure OA are still lacking. With the rise of tissue-cell engineering, stem cell therapy has gradually aroused great interest in treating OA. Cartilage stem/progenitor cells (CSPCs), a type of stem cell found on the surface of articular cartilage, have many similarities with mesenchymal stem cells (MSCs). These cells can be isolated and cultured from animals and humans and exist in articular cartilage over the body, such as the knee joint, patellofemoral joint, and temporomandibular joint. Due to their strong proliferative and chondrogenic differentiation abilities, CSPCs may contribute a lot to cartilage regeneration and repair in OA. We will provide an overview of the biological characteristics of CSPCs and their role in OA in combination with the research progress. Despite some existing limitations, CSPCs still offer an innovative idea for OA treatment with great advantages.

## 1 INTRODUCTION

Osteoarthritis (OA) is a common chronic degenerative joint disease closely related to factors such as age, gender and inflammation. The incidence of OA is mainly related to infection, injury, obesity, chronic strain, congenital malformation, genetics, hormones and other factors, but the pathogenesis has not been fully elucidated [1]. One of its salient pathological features is cartilage destruction. Articular cartilage is mainly composed of chondrocytes and extracellular matrix (ECM). In OA, initiation and progression, are mediated by various bioactive substances, such as pro-inflammatory and inflammatory cytokines, abnormal metabolism of chondrocytes, producing enzymes that lead to matrix degradation, chondrocyte apoptosis, and eventual cartilage destruction [2, 3]. Therefore, exploring methods to prevent cartilage degeneration and subchondral bone destruction is essential for OA treatment, among which activating joint regeneration with appropriate structures and functions may provide an ideal strategy.

Currently, the clinical treatment methods for OA mainly include drug therapy, intra-articular injection, physiotherapy, occlusal adjustment, and surgery, most of which are restricted to relieving symptoms. Meanwhile, new therapies, represented by regenerative and reparative therapy are constantly emerging. In recent years, a number of studies have found that when the cartilage is damaged, some stem cells play a certain role in chondrocytes replenishment and cartilage repair itself, indicating stem cell therapy has great potential in the treatment of OA [4, 5].

Cartilage stem/progenitor cells (CSPCs) are cells with stem cell properties isolated from the surface of articular cartilage. Due to the probable existence of a growth center on the surface of articular cartilage, Hayes *et al*. [6] firstly proposed that there may be a population of stem cells supporting cartilage growth on the surface of articular cartilage during their study of articular cartilage growth, which unlocked the research interest in this area. After that, Barbero *et al*. [7] discovered the presence of a population among chondrocytes that possess both clonal ability and differentiation potential for cartilage, osteogenesis, and adipogenesis. In 2004, Dowthwaite *et al*. [8] utilized differential adhesion to serum fibronectin as a sufficient measure to isolate and culture this kind of subpopulation with progenitor cell properties and did partial characterization.

At present, most scholars believe that these chondrogenic progenitor cells derived from mesenchymal stem cells (MSCs), have similar characteristics to MSCs, and can continue to complete cartilage differentiation as reserve cells for chondrogenesis. Alsalameh *et al*. [9] found an undifferentiated population in chondrocytes, showing a co-expression of cell surface markers CD105 (endogenous globulin) and CD166 (activated leukocyte cell adhesion molecule, ALCAM), which is the recognized combination used to define MSCs. So far, scholars have discovered a variety of biological characteristics and clinical application values of CSPCs. During the development of people's knowledge of chondrocyte progenitors, many nomenclatures have arisen, like cartilage stem cells (CSCs) [10], articular cartilage stem cells (ACSCs) [11], cartilage progenitor cells (CPCs) [12], fibrocartilage stem cells (FCSCs) [13]. At present, there is no definite evidence that these cells with different names do not belong to the same type of cells, nor can they be purified and separated from each other, so we temporarily define them as a type of cell group with similar characteristics and origins to express, described as cartilage stem/progenitor cells (CSPCs) in this review.

This review will focus on the important role of CSPCs in the occurrence and development of OA. It is aimed to summarize the biological behavioral characteristics of CSPCs in OA, and discuss the latest research progress on CSPCs. In addition, combined with existing studies, we will make an exploratory analysis of the therapeutic potential of applying CSPCs to OA, in terms of cartilage protection, chondrocyte regeneration, and inflammation inhibition.

## 2 BIOLOGICAL CHARACTERISTICS OF CSPCs

### 2.1 Markers and Identification of CSPCs

Cell labeling is an important method to identify and trace the role and behavior of a particular cell population in a complex multi-cellular system. By tagging stem cells, we can understand their distribution and trace their behaviors and outcomes. The following part mainly introduces the research progress of CSPCs labeling and tracing.

Currently, there are many techniques to obtain CSPCs from articular cartilage or non-articular cartilage. To isolate CSPCs from the cartilage tissue, differential adhesion to fibronectin, which Dowthwaite first used in 2004 [8], has been the most frequently used method. The basic process is to sort digested articular chondrocytes through fibronectin differential adhesion assay and then analyze their characterization to screen out stem cell populations with strong cloning ability [14, 15]. Although the products obtained through a differential adhesion assay to fibronectin may not be pure CSPCs, but a mixture containing chondrocytes [16], it is still proved to be the most practical technique to isolate CSPCs. Other techniques including clonogenicity [17], Perichondrium digest [18], and fluorescence-activated cell sorting [19] have also been proven to be equally effective in practical applications. Finally, the isolated populations can be confirmed based on their proliferation and differentiation abilities and specific genes or cell surface markers.

The tracer approaches of CSPCs also refer to those of other stem cells to a great extent. A common choice is to integrate the DNA of exogenous markers or nucleic acid analogues into the cell genome, so that the corresponding products can be expressed and identified. Green fluorescent protein (GFP) [20] and bromodeoxyuridine (BrdU) [21] are the most common exogenous markers, which are more suitable for long and short-term tracers, respectively [22]. Another method is to import markers directly into the target cells, including MRI contrast agents [23] and fluorescent dyes [24]. This method is less toxic to cells, but the markers decrease as cells divide, which may be less stable. Despite its good stability, exogenous markers have potential safety problems that may reduce stem cell viability or even cause malignant transformation, which may be related to gene integration [20, 21, 25]. In addition, there have appeared new technologies in recent years. In the past, it was considered that although iron oxide can label MSCs, chondrocytes and chondrogenic cells, it may affect the expression of chondrogenic genes, so it is mostly used in labeling MSCs [26]. While recently, Vinod [27] applied superparamagnetic iron-oxide (SPIO) to the labeling of CSPC and reported that, under the condition of controlled concentration of the marker, it will not affect the cell activity, proliferation and differentiation potential, and has the advantages of real-time simplicity and low cost. It has become an effective means of labeling CSPCs at present.

Early studies on the identification of CSPCs could only be preliminarily identified as “stem cell-like” cells by expressing MSCs-related surface markers. Criteria for the identification of MCSs formulated by the International Society for Cell Therapy (ISCT) include adherence, expression of specific cell surface markers (positive for CD105, CD73 and CD90, and negative for CD45, CD34, CD14 or CD11b, CD79a or CD19 and HLA-DR surface molecules), and the ability of tri-lineage differentiation capacity (adipogenic, osteogenic, and chondrogenic differentiation) [28, 29]. With the development of more studies, people's understanding of CSPCs identification method is gradually clear. This is due to reference and comparison of known chondrocyte and MSCs related genes and surface antigens. As an example, Ekram [30] reported that in their PCR results, CSPCs showed the expression of cartilage-related genes SOX9, TGF-β1, ACAN, BMP2 and GDF5, while MSCs did not express SOX9 and TGF-β1, reflecting the difference between CSPCs and MSCs. It showed that the markers of CSPCs and MSCs are similar to some extent, but also show some differences.

In recent years, single-cell analysis has greatly contributed to the accuracy of cell classification, identification and characterization. Single-cell level studies can provide a clearer description of the population characteristics of CSPCs. Ji *et al*. [31] sorted the cells in cartilage tissue by single-cell RNA-seq analysis, and proposed specific surface markers for CSPCs, including BIRC5, CENPU, UNE2C, DHFR and STMN1. Besides, by comparing the expression of 13 CSPCs surface markers using single-cell mass cytometry, Grandi *et al*. [32] revealed the existence of three different subgroups of CSPCs through 13 previously described markers for CSPCs, including CD49e, CD90 and CD166. These subgroups respectively stand for the subpopulations depleted in OA (CSPC I), the subpopulations unchanged in OA(CSPC II) and the subpopulations enriched in OA (CSPC III). They believed there was a CSPCs population with high regeneration ability and low inflammation correlation in articular cartilage. Thus, based on the development of single-cell technology, the characteristics of CSPCs have been provided with more details.

However, it is still difficult to take the exact isolation of MSCs, CSPCs and chondrocytes. Some scholars have been trying to find surface markers that can specifically differentiate CSPS. For example, Vinod *et al*. [33] has carried out experimental studies on CD49b and Kachroo *et al*. [34] did it on CD49e, but no reliable results have been obtained. Therefore, we collected some markers that are differentially expressed between CSPCs and MSCs (Table **1**) [10, 33, 35-42], as well as CSPCs and chondrocytes (Table **2**) [10, 35, 37, 43], hoping to provide clues for the discovery of specific CPSCs markers. In a word, the most likely markers that can be identified on the surface of CSPCs include CD9, CD29 (integrin β-1), CD73 (5'-nucleotidase), CD90 (Thy-1 membrane glycoprotein), CD105 (Endoglin), CD44, CD166, CD49e (integrin α-5), CD54 (intercellular adhesion molecule 1), as well as VCAM-1, STRO-1, and Notch-1 coexpressed in osteoarthritis, which provides important information for the continued in-depth study of CSPCs.

### 2.2 The Biological Characteristics of CSPCs

**Fig. (1):**
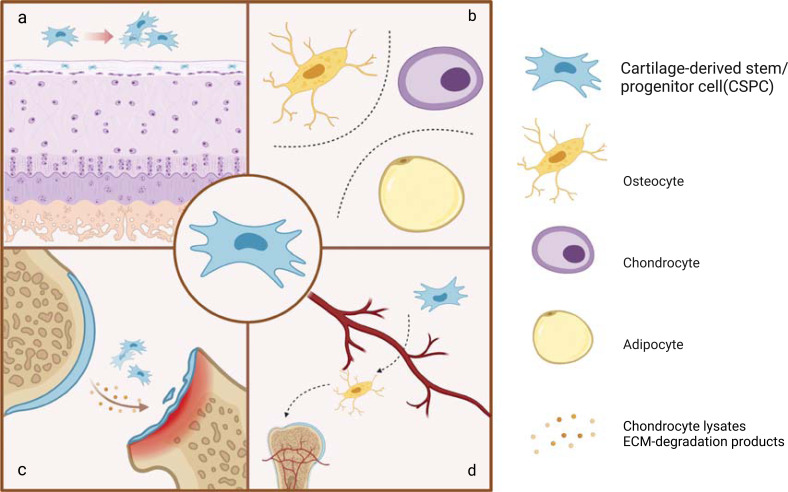
The biological characteristics of CSPCs. ⓐ ⓑ CSPCs, located in the superficial zone of articular cartilage, can proliferate and differentiate into chondrocytes, osteocytes and adipocytes. ⓒ CSPCs migrate to the cartilage defect area mainly under the induction of some biological substances, such as chondrocyte lysates and ECM-degradation products. ⓓ CSPCs interact with vascular endothelial cells to promote the regeneration of vascularized bone.

Articular cartilage can be divided into superficial, transitional and deep layers, each of which has a different cellular composition. In the experiments on cattle, horses and some other animals, researchers have found that such cell populations with stem cell characteristics could only be obtained from the cartilage surface [8, 44, 45], thus proving that CSPCs are located in the superficial zone of articular cartilage. Many studies have also found high expression of CSPCs markers (*e.g*., CD105 [46], CD166 [47], Notch-1 [48]) detected in the superficial areas of mature articular cartilage. This is also consistent with the CSPCs identification described above. These studies support the view that CSPCs exist on the surface of articular cartilage (Fig. **1**).

When CSPCs were first discovered, they received attention because of their clonality and tri-lineage differentiation potential [7]. Williams *et al*. [14] found that isolated CSPCs had longer telomere length and higher telomerase activity compared with chondrocytes, which enabled their strong proliferation ability to still exist after 60 passages *in vitro*. Later, Li *et al*. [49] verified that those cells expressing stem cell markers on the articular surface had strong cloning ability by using clonal genetic tracing and renewed through symmetrical division. Above all, the ability to differentiate is the most significant and also most concerning feature of CSPCs. Bi *et al*. [50] compared condyle-derived CSPCs with MSCs derived from the mandibular alveolar bone, and found that under the same induction and regulation, the two had similar adipogenic abilities, while CSPCs had the stronger chondrogenic ability and lower osteogenic ability. Embree *et al*. [51] further proved that CSPCs could spontaneously differentiate into chondrocytes and osteocytes to achieve endochondral ossification *in vivo*. Nathan *et al*. [52] found that the interaction between CSPCs and vascular endothelial cells also significantly promoted the osteogenic differentiation of CSPCs, leading to the neogenesis of vascularized bone. In short, CSPCs possess a strong ability for proliferation and multi-directional differentiation. These biological characteristics will be regulated by various factors such as cartilage ecological environment, intercellular interaction, cytokines, and gene expression. Subsequent studies on the behavior of CSPCs in disease development and therapeutic significance were also carried out based on their complex biological characteristics.

## 3 BIOLOGICAL BEHAVIOR REGULATION OF CSPCs IN OA

In the pathogenesis of OA, the destruction, apoptosis and regeneration of chondrocytes determine the development of the disease to a great extent. Since the discovery of CSPCs, their role in OA has been of great concern. Experiments in the literature focusing on human knee joint cartilage found that CSPCs were constitutively present in both normal articular cartilage and cartilage from OA patients. Furthermore, cell proliferation extent and stem cell markers expression in OA cartilage significantly increased [9], suggesting that CSPCs may be involved in the pathogenesis of OA. Though different mechanisms may cause OA in different joints, the role of CSPCs may involve joints in multiple parts of the body. Despite typical types of OA, temporomandibular osteoarthritis (TMJOA), as a special type of OA, is also included in the scope of this paper, and its related contents will be extra explained. The following will give an overview of the behavioral regulation of CSPCs in OA and discuss the mechanism of CSPCs participating in the disease process.

### 3.1 Recruitment of CSPCs in Cartilage Injury

The aggregation of CSPCs towards the cartilage-damaged area initiates their participation in OA. The chemotaxis of CSPCs is affected by a variety of biological and physical factors. Seol *et al*. [53] submitted that chondrocyte death is a key reason for the activation of CSPCs in OA. The cell lysates of damaged chondrocytes may be a strong chemoattractant for CSPCs. These lysates include high mobility group protein-1 (HMGB-1), IL-1β, insulin-like growth factor-1 (IGF-1), and platelet-derived growth factor (PDGF) [35], but it has not been determined that which factor plays a major role in aggregation events. Thereafter, this team found that the chondrocyte matrix could also promote CSPCs migration through ECM degradation enzymes [54]. Then, Joos [55] found that PDGF-BB and IGF-1 could induce CSPCs migration, while interleukin-1 β (IL-1β) and tumor necrosis factor α (TNF-α) inhibited this behavior. In addition to these biological factors, ultrasound can also lead to the chemotaxis of CSPCs [56], indicating that the migration of CSPCs may also be affected by physical factors. Similarly, in TMJ, condylar CSPCs were also observed to have this migration behavior, yet their migration ability was proved to be weaker than that of MSCs [50]. Although many PRG4+ cells were detected in the cartilage defect area, Decker *et al*. [57] observed that these cells proliferated in the synovial membrane instead of the cartilage surface layer. As a possible conjecture, Roelofs [58] speculated that some other stem cells might be involved in this kind of cartilage repair. Therefore, CSPCs are indeed recruited in OA, but how they colonize and multiple to initiate cartilage repair has not been definitively determined at present.

### 3.2 Some Genes have Significant Regulatory Effects on CSPCs

SOX9 gene is expressed in multiple organs and tissues throughout the body and has valuable functions in aspects of chondrogenesis, cartilage protection and cartilage repair [59, 60]. It has been confirmed to play a decisive role in the chondrogenic differentiation of CSPCs, which may promote CSPC-based cartilage compensation in OA. Compared with human MSCs, the mRNA level of SOX9 in CSPCs increased by 1.5 times [61]. Additionally, SOX9 was expressed at low levels of CSPCs in both adipogenic and osteogenic differentiation. In contrast, a high level of SOX9 was presented in chondrogenic differentiated CSPCs, accompanied by a high level of collagen type II, as well as a low level of collagen type I [10]. It can be preliminarily concluded that the chondrogenic differentiation ability of CSPCs is closely related to the expression of SOX9. Studies have found that when OA occurs, SOX9 loss can be detected in the damaged articular surface, which could be attributed to inflammatory cytokines [50, 62]. Recently, a study, especially for TMJOA shown, showed that the expression of SOX9 is closely correlated with the level and function of CSPCs [50]: *In vitro* culture mode, human-TMJ-derived CSPCs were observed to form cartilage microspheres with intact and continuous boundaries in chondrogenic induction medium. However, after the expression of SOX9 was down-regulated by shRNA, CSPCs could not form complete cartilage groups under the same culture conditions and significantly decreased chondrocyte-related protein expression. *In vivo* experiments, after injecting cells into the cartilage defect area, it was observed that the repair ability of SOX9-knockout CSPCs was significantly reduced [50]. As an important regulator of chondrogenesis, the expression of SOX9 simultaneously regulates chondrocytes and CSPCs in OA, which is an important basis for CSPCs to participate in the pathogenesis of OA.

In addition to SOX9, some other genes are also important to CSPCs. Compared with MSCs, CSPCs overexpressed PRG4(lubricin) and chondrocyte-related genes S100A1 and S100B [56]. PRG4 can not only be used as an important means to identify and track the distribution of CSPCs and prove the ability of CSPCs to differentiate into chondrocytes, but may also promote CSPCs to secrete lubricin to protect joint surface. The expression level of S100A1 in chondrocytes is extremely closely correlated with SOX9, and has been reported to be down-regulated in OA, suggesting that S100A1 has a probable connection with OA [63]. Overexpression of S100B in cartilage tissue inhibits inflammatory responses related to IL-1β and TNF-α in the synovium of OA joint, thereby affecting the repair of cartilage injury [64]. Another study revealed that S100A1 and S100B were expressed homogeneously in all cartilage zones and decreased during dedifferentiation [65]. These studies suggested that CSPCs possibly have an increasing number and play a role during OA. The latest research showed some other genes, such as KCNMA1 and KCNN4, which encode members of the calcium-activated potassium channels (KCa) are more strongly expressed in CSPCs than in MSCs [12]. And it turns out that KCa plays a role in maintaining the phenotype of chondrogenic precursor cells. In addition, some genes also have important effects on the migration and recruitment of CSPCs. For example, the expression of CXCL12 significantly increases the chemotaxis of CSPCs [56], while MicroRNA-375 inhibits this migration [66]. These studies add key evidence to the difference and connection between CSPCs with MSCs and chondrocytes at the gene level, providing some enlightenment for us to further study the role of CSPC in OA.

### 3.3 The Fate of CSPCs During OA is Regulated by Multiple Signaling Pathways

#### 3.3.1 TNF-α/Nf-κB Signaling

The pathogenesis of OA reflects the participation of various inflammatory factors and signaling pathways. TNF-α and IL-1β, as the main pro-inflammatory cytokines released in the joint after OA occurs, activate the transcription factor nuclear factor-κB (Nf κB) in chondrocytes. Translocation of NF-κB into the nucleus induces the expression of different gene subsets encoding inflammation, apoptosis, and the release of ECM degradation enzymes [67]. In cartilage, TNF-α/Nf-κB can degrade the cartilage matrix by promoting the secretion of matrix metalloproteinase (MMP) and other pro-inflammatory factors from fibroblasts, meanwhile, inhibiting chondrogenesis and increasing apoptosis by down-regulating SOX9, resulting in cartilage destruction [68, 69]. Some scholars have explored the role of TNF-α in the fate of CSPCs based on TNF-α/Nf-κB signaling: Tong [11] demonstrated that activation of Nf-κB pathway inhibited the reaction of CSPCs during OA, while Nf-κB inhibitors reversed this phenomenon, thereby protecting cartilage tissue and inducing cartilage regeneration.

In terms of the specificity of TMJ, Yuan *et al*. [70] found that inhibiting the Nf-κB pathway activated by inflammatory factors such as TNF-α could reverse TMJOA progression. Bi [71] found that TNF-α inhibited the survival, proliferation and differentiation of CSPCs through the Nf-κB pathway, and promoted their apoptosis, while TNF-α inhibitors could effectively prevent this effect. They observed that etanercept, a TNF-α inhibitor, improved the chondrogenic properties of CSPCs, promoted chondrogenic differentiation of CSPCs, and alleviated cartilage degradation of TMJOA. Since Nf-κB is a common pathway through which multiple inflammatory factors function, inhibition of other inflammatory factors, such as the topical application of PDGF-BB and IGF-1 acting as anti-inflammatory agents by inhibiting IL-1β-mediated Nf-κB signaling [72], may also play a similar role in cartilage protection.

Overall, Nf-κB signaling pathway plays an important role in regulating the biological behavior of CSPCs, and inhibition of this pathway may help CSPCs participate in the repair of OA.

#### 3.3.2 Notch Signaling Pathway

Notch gene expression products exist widely in the body, and the signaling pathway mediated by Notch plays an important role in cell genesis, development, proliferation and differentiation. Notch signaling pathway is composed of Notch receptors (Notch1-4), Notch ligands (delta-like1, 3, and 4,Jagged 1 and 2), transcription factors, regulatory factors, and target molecules (Hes, Hey) [73, 74], which regulate the occurrence and outcome of chondrocytes, as well as the differentiation of upstream stem cells to affect chondrogenesis [75]. Previous studies have shown that the abnormal activation of Notch in articular cartilage contributes to the development of OA [76-78]. In addition, some research data suggest that increased Notch level or activity is positively correlated with the severity of OA after joint trauma [79], suggesting that Notch is involved in the pathological process of OA. Recently, Ruscitto [80] firstly proved that Notch's regulation in CSPCs fate: Notch activation can promote osteogenic and chondrogenic differentiation of CSPCs, but inhibit adipogenic differentiation. With inflammation, Notch activation promotes cartilage-bone transformation and osteogenic differentiation of CSPCs, thereby damaging the normal joint structure.

In terms of TMJ, Lan *et al*. [81] detected a noteworthy increase in the expression of Notch1, Jagged1 and Hes5 in the condylar cartilage of TMJOA mice through immunohistochemistry, indicating the activation status of Notch signaling in TMJOA. Later, Luo *et al*. [82] also observed that Notch related markers increased in TMJOA animal model, and Notch signaling inhibitor DAPT could delay the TMJOA process.

However, the role of Notch signaling in OA is somewhat contradictory, and its role in promoting chondrogenic differentiation of CSPCs in OA has not been fully elucidated. Anyhow, the Notch signaling pathway is significantly activated in OA, which can influence the differentiation direction of CSPCs and affect disease development through multiple mechanisms.

#### 3.3.3 WNT Signaling and Other Related Pathways

WNT proteins signal through canonical WNT/β-catenin pathway and other non-canonical pathways (WNT/PCP, WNT/Ca^2+^, *etc*.), and participate in multiple physiological processes such as joint formation, bone tissue stability, and the pathology of many diseases [83]. After the occurrence of OA, WNT signaling affects the regeneration and cartilage repair ability of CSPCs. Much evidence have shown that overactive WNT signaling can lead to degeneration and depletion of CSPCs [84, 85]. Blocking WNT signaling with inhibitor SOST could boost chondrogenic differentiation of CSPCs, maintain the number of CSPCs, and contribute to CSPCs-based cartilage repair [51]. However, recent studies have shown that enhanced WNT signaling is detected in the cartilage-damaged area of the joints, which antagonizes cartilage damage induced by IL-β and recruits CSPCs to compensate for cartilage loss. This effect may occur through the canonical WNT/β-catenin and non-canonical WNT/ JNK-cJUN pathway [86]. This also indicates that different WNT pathways may have different effects on arthritis, and both excessive activation and excessive suppression can lead to cartilage loss [86].

In addition, Hippo/YAP pathway,which presents multiple interactions with WNT pathway has also been shown to affect the proliferation and differentiation of CSPCs. According to Qin’s finding [87], the effects of suppressing YAP included downregulating the proliferation activity of CSPCs, promoting apoptosis of CSPCs and blocking self-renewal of CSPCs, generally causing damage to stem cell pool on the surface of articular cartilage. Consequently, cartilage damage was aggravated. Since YAP not only relies on the Hippo pathway to function but also acts as a regulatory factor of the WNT pathway [88], the concrete mechanism of YAP regulation of CSPCs has not been determined yet.

In summary, WNT signaling shows complex characteristics in the regulation of CSPCs. Some particular WNT pathways may promote the cartilage repair of CSPCs and maintain the stability of CSPCs pool. However, each type of WNT pathway may have different functions. Thus the specific regulatory mechanism is worth further exploring.

## 4 THERAPEUTIC POTENTIAL OF CSPCs IN OA

**Fig. (2):**
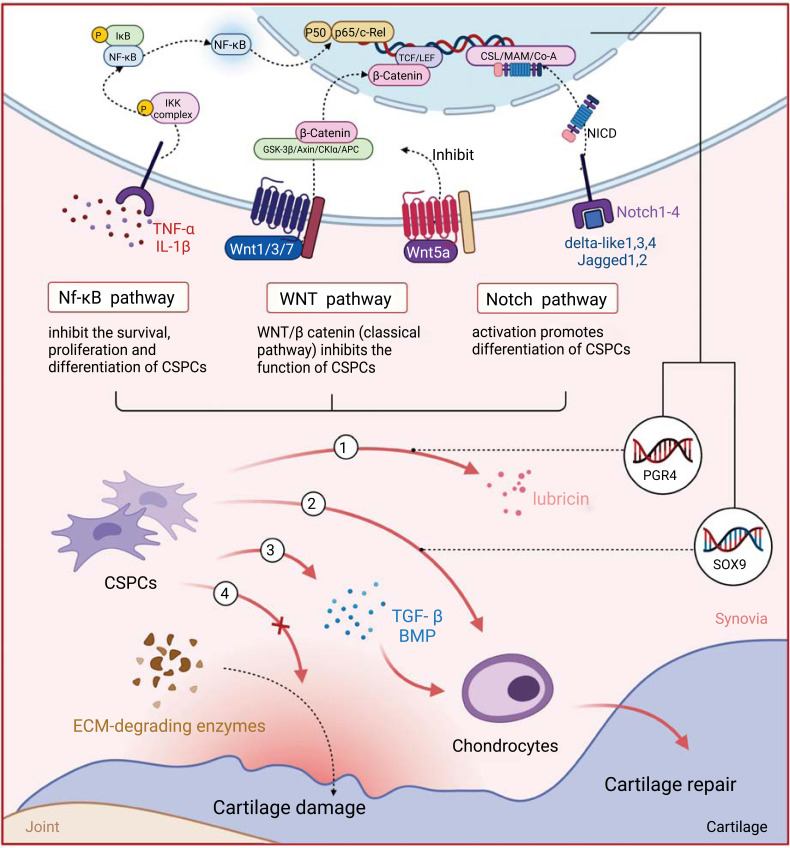
Therapeutic potential of CSPCs in OA. ① CSPCs can produce lubricin, which can lubricate the surface of joints and reduce the internal friction of joints. ② CSPCs have strong chondrogenic differentiation potential to compensate for cartilage loss. ③ ④ CSPCs can protect both chondrocytes and cartilage matrix through producing cytokines and inhibiting the release of ECM degradation enzymes. Some signaling pathways and specific gene expression have impact on the proliferation, differentiation and function of CSPCs. Developing drugs targeting these pathways may promote OA treatment.

With the rapid development of cellular engineering, tissue engineering and regenerative medicine, stem cell therapy has become a promising therapeutic with high efficacy and development potential. When special stem cell populations with a strong ability to differentiate into chondrocytes were discovered, the therapy was introduced as a treatment for OA (Fig. **2**). Studies have found that MSCs isolated from diverse tissue, containing bone marrow, adipose tissue and umbilical cord blood, had good performance in OA treatment [89]. Because of this, CSPCs with similar biological properties to MSCs are likely to have similar therapeutic capabilities. The successful application of MSCs laid a good foundation for the attempt of CSPCs therapy and provided a rich experience for treating OA by CSPCs.

### 4.1 Possible Therapeutic Mechanisms of CSPCs

The therapeutic potential of CSPCs can be summarized into two aspects. One is the regeneration and repair of damaged joints. It has been detailed above that CSPCs participate in the pathogenesis of OA through a variety of complex mechanisms, accordingly having the possibility of treating OA. The most important thing about this therapy is the strong chondrogenic differentiation potential of CSPCs. As for clear evidence, intra-articular injection of CSPCs into TMJOA model rats significantly delayed the degradation of articular cartilage, and it was observed through lineage tracing that CSPCs could directly repair articular cartilage [61]. In another study, CSPCs were used as seed cells to repair full-layer bovine femoral cartilage defects, and it was found that the regenerated cartilage had sufficient physiological characteristics and mechanical properties [90]. Moreover, compared with MSCs, CSPCs not only have higher chondrogenic potential but also naturally exist in the superficial layer of articular cartilage. Therefore, CSPCs therapy may directly initiate *in-situ* self-repair of articular cartilage by certain means [11]. The differentiation of CSPCs is regulated by multiple signalings like Nf-κB, Notch and WNT, and the initiation of cartilage regeneration depends on the activation or inhibition of the corresponding signaling. That means the therapy might be able to avoid surgery but simply by regulating the target signal with drugs.

Another aspect is to delay disease progression and relieve symptoms, in which the secretion and regulation of lubricin play an important role. Lubricin, as a glycoprotein in synovial fluid, can lubricate the surface of joints and reduce internal friction, which is an intra-articular injection therapy for OA itself [91, 92]. The encoding gene PGR4 of lubricin is highly expressed in CSPCs [56, 93], thus, the aggregation of CSPCs can produce a large amount of lubricin, reshape joint surface, and achieve the purpose of treating OA. This treatment pathway not only requires the recruitment and proliferation of CSPCs, but also the activation of some signals to promote the release of lubrication. Wei *et al*. [94] found that PGR4 expression was down-regulated in OA, and the activation of the epidermal growth factor receptor (EGFR) could reverse this phenomenon and retain the expression of PGR4 in articular cartilage, so that improving the lubrication function of articular cartilage. Xuan *et al*. [95] found that specifically enhancing β-catenin in CSPCs could promote the expression of lubricin in mice joints and delay the progression of OA, indicating that classical WNT/β-catenin signaling could not only regulate the differentiation of CSPCs, but also influence the production of lubricin, which is another important means for OA treatment.

Delaying the progression of the disease also depends on the protective effect of CSPCs. CSPCs have the function of protecting chondrocytes and cartilage matrix. CSPCs can produce transforming growth factor β (TGF-β) and bone morphogenetic protein *in vitro*, and these signaling molecules act on chondrocytes to promote chondrocyte re-differentiation and maintain ECM stability [10]. At the same time, CSPCs can also inhibit the release of MMP and other enzymes that destroy the cartilage matrix, therefore reducing the loss of cartilage matrix [35]. From these results, we can indicate that CSPCs prevent OA progression by stabilizing chondrocyte microenvironment and inhibiting chondrocyte death.

Recently, some animal experiments based on CSPCs for the treatment of joint and cartilage-related diseases have been reported. Vinod *et al*. [96] injected CSPCs into a rabbit knee OA model, and found less cartilage wear, less osteophyte formation, and synovial thickening at the joint than what they were before treatment. Ekram *et al*. [30] used CSPCs differentiated from human umbilical cord-derived MSCs for the study of intervertebral disc degeneration in rats. It was found that compared with MSCs, CSPCs could improve intervertebral disc degeneration and reduce pain and inflammation. These studies have shown that CSPCs have considerable therapeutic effects on joint inflammation and degenerative diseases.

### 4.2 Clinical Application Methods and Recommendations

In addition to CSPCs' inherent therapeutic potential, there are numerous factors to consider. For the therapeutic application of CSPCs in the treatment of osteoarthritis, a suitable stem cell niche is necessary. To obtain the best healing response, growth factors, biocompatible scaffolds, and small interfering RNA (siRNA) can help CSPCs adhere and grow, stimulate chondrogenesis, and promote cellular functioning [97].

As for scaffolds, in chondrogenesis generated by platelet-rich plasma (PRP), CSPCs showed greater cell density than chondrocytes and greater chondrogenic capacity than MSCS [98]. CSPCs with PLGA scaffold, considered another good choice, demonstrated the capacity to stimulate osteochondral regeneration in the rabbit knee, which is helpful for repairing more complicated tissues like osteochondral [99].

Additionally, there are a number of other strategies that help CSPCs work. Exosomes from chondrocytes can offer ectopic implanted CSPCs a suitable habitat, increasing the stability of the resultant cartilage and inhibiting hypertrophic differentiation and vascular growth [100]. Fibronectin (FN) can enhance the proliferation, migration, and chondrogenesis of CSPCs, and can be targeted to activate CSPCs for the treatment of cartilage injury [101]. By injecting into the joint, it may be feasible to encourage the migration of CSPCs from healthy tissue to the wounded region while avoiding the risks associated with open surgery. Hypoxic extracellular vesicles derived from hypoxic adipose-derived stem cells (ADSCs) induce CSPCs to produce more cartilage matrix and proteoglycans [102]. The activation of CSPCs is also affected by mechanical forces, which stimulate CSPCs' repair capability under an appropriate amount of loading. According to a recent study, the mechanobiological effects of radial extracorporeal shock wave (R-ESW) can significantly increase the expression of proteoglycan and collagen II, as well as the proliferation of CSPCs [103]. All of these can be used as important auxiliary means in clinical application of CSPCs.

Observing cell survival and distribution following transplantation is crucial for investigations on efficacy and mechanism. A recent study reported that tri-modal *in vivo* imaging of subcutaneous CSPCs utilizing upconversion nanoparticles (UCNPs) allows for more precise cell counting. Although there are still imaging flaws and room for improvement in nanoparticle performance, there is currently a considerable clinical application value [104].

Regarding the choice of patient population, not every member of the population can treat OA with CSPCs. The patient's ability to withstand the procedure should be taken into account first. Therapy of OA by activating endogenous CSPCs is only effective for the repair of minor damage, while exogenous CSPCs need to be supplemented with tissue engineering technology on account of the poor targeting and short half-life [105]. As people get older, their ability to heal cartilage declines [106]. Clinical cases of CSPCs use in younger patients have been documented, and all of these patients reported good quality of life without any major problems [37]. However, there are still few pertinent researches involving elderly. Moreover, although stem cell therapy has been recognized as a safe treatment, therapies based on CSPCs lack sufficient clinical application data to confirm safety, which means further experimental exploration is needed in this area.

Summary of various aspects, CSPCs can realize OA treatment through the composite mechanism of protecting cartilage, inhibiting cartilage degeneration, promoting cartilage regeneration and secreting joint lubricin. Although still lack substantial clinical trial results to support, CSPCs have shown good potential in OA treatment and may become an excellent therapy to cure OA in the future.

## CONCLUSION

The traditional treatment of OA focuses on relieving pain, stopping disease progression, and improving joint function. Currently, how to retard the irreversible degradation of cartilage and achieve cartilage repair in OA still lacks effective interventions. The therapies of OA still need innovation and breakthroughs. Stem cell therapy based on CSPCs, a group of stem-like cells having advantages of proliferation, chemotaxis, and great potential of differentiating into chondrocytes, can become a meaningful method to treat OA. CSPCs have been proved to play an important role in the pathogenesis of OA, which can protect cartilage, inhibit degeneration of articular cartilage and promote regeneration of articular cartilage through multiple ways, thus possessing extraordinary therapeutic potential. With the help of histiocytic engineering and some activating factors, CPSCs can migrate to the damaged site, efficiently complete cartilage repair, relieve symptoms and improve prognosis. Compared with traditional drug treatment, this method can reverse the disease process and realize joint remodeling. Compared with joint replacement and other surgical methods, this method is more minimally invasive and has a wider application range. Compared with MSCs-based stem cell therapy, CSPCs have higher chondrogenesis potential. At present, this method has initially begun to enter clinical practice, and has achieved certain results. Although further exploration is needed, we must admit that the future of this therapy is very promising.

## Figures and Tables

**Table 1:** Comparison of CSPCs marker expression with MSCs*.

**Surface Markers**	**CD9**	**CD13**	**CD18**	**CD29**	**CD31**	**CD44**	**CD49e**	**CD54**	**CD73**	**CD90**	**CD105**	**CD106**	**CD146**	**CD166**	**CD271**	**Notch-1**	**STRO-1**
MCSs	+	+	+	+	+	+	+	+	+	+	+	+	+	+	+	+	+
CSPCs	+	+	-	+	-	+	+	+	+^higher^	+^higher^	+^higher^	+	+	+	-	+	+^lower^

* +: Positive. -: Negative. +^higher^/+^lower^: The expression level is higher/lower by contrast.

**Table 2:** Comparison of CSPCs marker expression with Chondrocytes*.

**Surface Markers**	**CD13**	**CD29**	**CD44**	**CD73**	**CD90**	**CD105**	**CD106**	**Notch-1**	**STRO-1**	**COL2**
Chondrocytes	+	+	+	+	+	+	+	+	+	+
CSPCs	+^higher^	+^lower^	+^higher^	+^higher^	+^lower^	+^lower^	+^lower^	+^lower^	+	+^lower^

* +: Positive. -: Negative. +^higher^/+^lower^: The expression level is higher/lower by contrast.

## LIST OF ABBREVIATIONS

OA: Osteoarthritis
MSCs: Mesenchymal Stem Cells
ECM: Extracellular Matrix
CSPCs: Cartilage Stem/Progenitor Cells
CSCs: Cartilage Stem Cells
ACSCs: Articular Cartilage Stem Cells
CPCs: Cartilage Progenitor Cells
FCSCs: Fibrocartilage Stem Cells
GFP: Green Fluorescent Protein
TMJOA: Temporomandibular Osteoarthritis
